# My digital mentor: a mixed-methods study of user-GAI interactions

**DOI:** 10.3389/fpsyg.2025.1636480

**Published:** 2025-11-18

**Authors:** Lei Xian, Guangqiu Cao, Na Zhang

**Affiliations:** 1 School of Management, Xiamen University, Xiamen, China; 2 School of Management, Xiamen University Tan Kah Kee College, Zhangzhou, China

**Keywords:** generative artificial intelligence (GAI), online learning, infusion use, mixed-method, configurations

## Abstract

**Introduction:**

Generative Artificial Intelligence (GAI) has emerged as a powerful tool in online learning, offering dynamic, high-quality, and user-friendly content. While previous studies have primarily focused on GAI’s short-term impacts, such as users’ acceptance and initial adoption, a notable gap exists in understanding long-term usage (i.e., infusion use) and the psychological mechanisms.

**Method and results:**

This study employs a two-stage mixed-methods approach to investigate users’ infusion use of GAI in online learning scenarios. A semi-structured interview (*N* = 26) was conducted in the first stage to develop a systematic framework of influencing factors. These factors include intelligence, explainability, response time, integrability, accuracy, source credibility, personalization, and emotional support. The second stage empirically validated the research framework using survey data of 327 participants. We find that the eight factors influence users’ infusion use through two key psychological mediators: perceived value and satisfaction. We also used the fsQCA method to obtain the configurations. These configurations demonstrate that no single factor alone is sufficient; rather, it is the combination of multiple factors that fosters users’ infusion use.

**Discussion:**

Our findings contribute to expanding the literature on the application of the theoretical literature on technology adoption in online learning contexts and provide practical implications for developing effective user-GAI interaction.

## 1 Introduction

Generative Artificial Intelligence (GAI) like ChatGPT simulates human cognitive processes through deep learning, extensive training datasets to generate innovative content (Yuan et al., 2024). In online learning, GAI serves diverse roles, such as virtual teachers, teaching assistants, and automatic grading (Liang et al., 2023; Peng and Wan, 2024). It supports real-time Q&A, tracks learning progress, and facilitates speaking practice through interactive dialogue (Du and Lv, 2024; Shao et al., 2025). One example is Quizlet Q-Chat, which adapts to different students’ learning habits and helps them master key concepts through customized Q&A sessions.^1^

Unlike traditional offline and online classes, GAI overcomes temporal and spatial constraints, enabling learners to access knowledge anytime and anywhere. This 24/7 learning support benefits non-student groups, such as working professionals. GAI technology can provide these individuals with flexible access to knowledge, enabling them to acquire cutting-edge industry skills and knowledge rapidly. It engages users through a communication that closely mimics human-to-human interaction. GAI can deeply understand users’ needs and preferences and provide them with a customized learning approach. The technology’s adaptability facilitates seamless integration into daily routines. It also encourages comprehensive utilization of its functional capabilities. However, existing literature lacks an exploration of the key factors driving users’ infusion use in online learning contexts.

Infusion use refers to users’ profound integration of information technology/systems (IT/S) into their daily learning processes to maximize technological potential (Chen et al., 2021; Hassandoust and Techatassanasoontorn, 2022). It represents the ultimate state of technology adoption, i.e., when the technology is fully embedded in the users’ daily lives (Jones et al., 2002). Existing research has extensively investigated users’ long-term usage behaviors, such as continuous usage and deep use. Continuous usage primarily concerns IT adoption and long-term usage decisions (Bhattacherjee, 2001), yet it does not fully capture the nature of post-adoption behavior (Hu et al., 2024). Deep use, on the other hand, examines the extent to which users leverage IT to achieve personal goals (Ogbanufe and Gerhart, 2020). However, these post-adoption behaviors represent efforts toward achieving the ultimate state of infusion use, which reflects the optimal alignment among users, IT, and tasks (Hu et al., 2024).

In this study, infusion use refers to users’ active, repetitive, and long-term in-depth use of GAI. This study focuses on infusion use for three reasons. First, unlike traditional AI, infusion use requires users to adopt an open attitude toward GAI, deeply understand its functions, and actively explore its potential, imposing higher users’ demands (Hu et al., 2024). Therefore, it may be challenging for users to realize the full potential of the GAI. Second, existing studies focus on short-term behaviors such as users’ acceptance (Wong et al., 2023; Li Y. et al., 2024), adoption (Chang and Park, 2024; Pathak and Bansal, 2024), and intention to use (Kim et al., 2021; Camilleri, 2024) of AI technologies, while neglecting users’ long-term behaviors (i.e., infusion use). In contrast to short-term use, infusion use emphasizes continuity and regularity, focusing on users’ ability to use GAI to support their learning, which is essential for creating superior business value (Chen et al., 2020; Chen et al., 2021). As a result, infusion use is considered a more promising pattern for technology adoption (Hu et al., 2024). Third, most studies have employed theoretical frameworks such as the technology acceptance model (TAM) (Zou et al., 2025; Kim et al., 2025), the unified theory of acceptance and use of technology (UTAUT) (Wang, 2025; Xu et al., 2025), the theory of planned behavior (TPB) (Al-Emran et al., 2024), and the task-technology fit (TTF) (Du and Lv, 2024) to investigate users’ usage behavior of GAI. However, few scholars have systematically explored GAI’s long-term use (i.e., infusion use) from a comprehensive perspective. As an emerging technology, the key factors influencing users’ infusion use of GAI have not been sufficiently explored. Therefore, it is essential to analyze the key factors and frameworks that drive the widespread infusion use of GAI.

Based on the above analysis, this study develops the following research questions:

(1) *Which factors influence users’ infusion use of GAI?*
(2) *What are the influencing mechanisms of these factors on infusion use?*
(3) *What are the configurational effects of these factors on infusion use?*

To answer these questions, a two-stage methodology was employed. Stage 1 qualitative research, this study collected data from 26 users through semi-structured interviews to develop a comprehensive theoretical framework to understand the factors influencing users’ infusion use of GAI in online learning contexts. In stage 2, we conducted a quantitative study that empirically validated the research framework using 327 participants’ survey data. At last, fuzzy set qualitative comparative analysis (fsQCA) methods were integrated to analyze all samples, validating the configurational effects.

This study makes unique contributions to the literature. First, we expand the theoretical understanding of GAI in online learning scenarios by developing and empirically validating a research framework that systematically explains how GAI’s characteristics influence infusion use. Second, we verify the critical mediating roles of perceived value and satisfaction in the behavioral formation process, thereby gaining insights into users’ psychological mechanisms. Finally, our findings offer actionable guidance for GAI educators, technology developers, and policymakers to enhance technology integration and maximize educational impact.

## 2 Theoretical background

### 2.1 SOR model

The Stimulus-Organism-Response (SOR) model was proposed by Mehrabian (1974), as a theoretical framework for exploring the relationship between external stimuli and organismic responses. This model posits that behavioral performance is not merely a stimulus–response paradigm, but rather, it is achieved through cognitive processing by the organism to elicit a specific response. Specifically, stimuli (S) is defined as the various types of external factors in the environment that influence the internal mental state or cognitive processes of the organism (O), ultimately leading to a specific behavioral response (R). These stimuli activate various internal processes, including cognition, affect, and evaluation, ultimately determining the organism’s behavioral response (Xie et al., 2023). The concept of an organism’s perception establishes a link between stimulus and response, thereby explaining the process by which an organism is stimulated and responds.

While established models like TAM effectively explain technology acceptance through perceptual factors like usefulness and ease of use (Al-Adwan et al., 2023; Zou et al., 2025), the SOR model offers a more comprehensive perspective. It captures not only external technological characteristics but also the crucial mediating role of users’ internal states—particularly their cognitive processing (Fu et al., 2025). This model provides a more nuanced understanding of how external environmental stimuli interact with individual cognition and evaluation to shape behavioral responses (Chen, 2023; Liu Y. F. et al., 2023). Consequently, the SOR model is increasingly applied within online learning, including e-learning platforms (Fu et al., 2025), AI teaching assistants (Peng and Wan, 2024), and mobile-assisted language learning (Lee and Xiong, 2023).

This range of applications demonstrates the SOR model’s validity for GAI online learning contexts. It not only aids in understanding how GAI features (stimuli) and users’ internal psychological processes (organism), but also provides actionable recommendations for optimizing the GAI online learning experience and enhancing users’ infusion use.

### 2.2 The mixed-methods approach design

**Figure 1:**
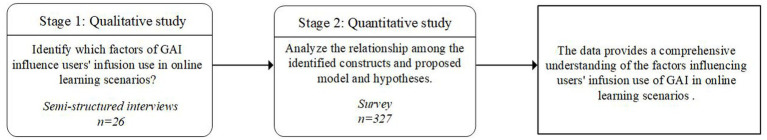
Mixed-method design.

Our study employed a mixed-methods approach to explore the infusion use of GAI in online learning scenarios, following established methodological guidelines (Venkatesh et al., 2016; Creswell and Creswell, 2018). The steps for an exploratory sequential design are as follows: First, the qualitative stage involved 26 semi-structured interviews to explore factors influencing users’ infusion use of GAI. We proposed our research model and hypotheses based on the results of stage 1. Then, stage 2 tested the hypotheses through an online survey. This study is conducted in two stages, as illustrated in Figure 1.

The mixed-methods approach is particularly appropriate for our study. First, this design offers advantages over single-method approaches by simultaneously addressing both confirmatory and explanatory research questions (Creswell and Creswell, 2018). It aligns perfectly with the dual nature of our investigation. Second, the application of GAI in online education context presents a degree of novelty, making it difficult for existing theories to provide a thorough description and explanation of the issues (Venkatesh et al., 2016).

## 3 Stage 1: the qualitative study

### 3.1 Data collection

This study employed semi-structured interviews conducted through face-to-face interviews and online video conferences. The research group first screened participants with prior experience using GAI, who usually use GAI as their main learning tool and can express their opinions based on their experiences. Participants were recruited using purposive sampling techniques to ensure their suitability for the study topic. All participants were requested to indicate their willingness to participate in semi-structured interviews. Among them, 13 were female (50%). The average age of participants was 29.77 (SD = 8.75). Most participants were young and middle-aged individuals under 35, aligning with QuestMobile’s finding that AI users are predominantly concentrated in this age group.^2^

Before the interviews, the researcher established several guidelines with participants, including encouraging open sharing, preventing interruptions, and ensuring the anonymity of all information, to foster an open and secure communication environment. During the interviews, the researcher initially collected basic demographic information and explained GAI. Participants were then asked to describe specific examples of using GAI for learning. The interviews focused on the role of GAI in the participants’ learning, their most memorable experiences, challenges encountered in using GAI, and the subsequent impacts. The detailed interview protocol is shown in Appendix A.1. Each interview lasted 20–30 min, with all participants agreeing to audio recording. After the interviews, the audio recordings were transcribed verbatim into textual material for subsequent qualitative analysis. Data were collected between December 2024 and January 2025. Interviews ceased upon reaching data saturation, defined as the point when no significant new information emerged from the data (Shao et al., 2024). Demographic details of the participants are presented in Table 1.

**Table 1:** Participants’ basic information.

No.	Age	Gender	Profession	No.	Age	Gender	Profession
P1	19	Female	Student	P14	28	Female	Pre-school teacher
P2	20	Male	Student	P15	28	Male	Student
P3	21	Female	Student	P16	30	Female	Student
P4	22	Male	Student	P17	30	Male	Student
P5	23	Male	Chemical inspector	P18	31	Female	High school teacher
P6	23	Male	Student	P19	32	Female	College teacher
P7	24	Female	Student	P20	33	Male	Interior designer
P8	24	Male	Student	P21	35	Male	AI engineer
P9	24	Female	Student	P22	39	Female	University teacher
P10	25	Female	E-commerce operator	P23	42	Male	Manufacturing engineer
P11	25	Female	Student	P24	45	Female	Accountants
P12	26	Male	Financial analyst	P25	48	Male	Self-employed
P13	26	Male	Student	P26	51	Female	Psychological Counselor

### 3.2 Data analysis

First, the semi-structured interview transcripts were pre-processed to remove content unrelated to GAI in online learning scenarios. Second, semantically ambiguous or irrelevant content was eliminated. Next, responses reflecting interviewees’ misinterpretation of the questions were excluded. Finally, the transcripts were coded and labeled line-by-line according to the logical sequence of the interview content. According to Liu Y. L. et al. (2023), we adopted thematic analysis combining “top-down” framework analysis (Ritchie and Spencer, 2002) and “bottom-up” grounded theory (Glaser and Strauss, 2017). We analyzed the interview data by coding, theming, decontextualizing, and recontextualizing.

This study utilized NVivo 11.0 software to conduct a thorough analysis of the interview data, examining the content word-by-word, sentence-by-sentence, and paragraph-by-paragraph. The researchers were instructed to use original phrases from the interview transcripts for labeling during the coding process. Two graduate students subsequently organized the data based on the initial nodes. Throughout this process, the original information was continuously compared and revised, with all meaningful themes and concepts being precisely extracted.

To minimize researcher bias in the coding process, two graduate students independently coded the data through a back-to-back approach. Both coders were native Chinese speakers and familiar with GAI. Before starting the coding process, a centralized meeting was conducted to align the coders on the procedures and clarify relevant concepts and theories. After each coding round, the results from both coders were compared, the same initial concepts were merged, and the coding conflicts were discussed with experts. Finally, the concepts were summarized and reorganized. After repeated discussions, only concepts agreed upon by both coders were retained. The complete coding process is presented in Appendix A.2.

### 3.3 Findings from interviews

Through semi-structured interviews, we identified several factors influencing the infusion use of GAI, such as intelligence, explainability, response time, integrability, accuracy, source credibility, personalization, emotional support, perceived value, and satisfaction. Building upon these exploratory findings, we subsequently designed a quantitative study to empirically validate the hypothesized relationships between these variables. Analysis of interview text reveals that users primarily employ ChatGPT, Doubao, ERNIE Bot, Deepseek, Gemini, Kimi, and other GAI platforms, reflecting the diversity of GAI tools within online learning contexts.

## 4 Stage 2: the quantitative study

### 4.1 Development of hypotheses

Intelligence reflects GAI’s capability through environmental perception, adaptive learning, problem-solving, and goal attainment. This characteristic of continuous evolution through feedback leads users to recognize it as a genuine intelligence (Moussawi et al., 2023). As the most critical factors of AI technology (Bartneck et al., 2009), intelligence fundamentally relies on natural language processing technologies that enable AI to simulate human cognitive processes in language comprehension and production (McLean et al., 2021). The statement is echoed by the qualitative investigation. For example, interviewee (P6) mentioned: “*Because I think ChatGPT records all my previous conversations, I think its answer will be more professional and more in line with my heart.*” GAI exemplifies this intelligence through its extensive knowledge repository and professional response capabilities, delivering not merely accurate and compelling answers but also formulating solutions that satisfy users through concise and coherent language outputs (Priya and Sharma, 2023). During interactive processes, GAI exhibits a high level of attentiveness to the users’ needs while employing diverse response strategies, which significantly enhance users’ experience and foster positive attitudes toward the technology.

Priya and Sharma (2023) pointed out that intelligence manifests in three critical dimensions of information generation: effectiveness, efficiency, and reliability. These dimensions not only constitute the fundamental drivers of GAI advancement but also serve as critical factors shaping users’ perceptions. As demonstrated in some studies, a direct relationship between GAI’s intelligent performance and its functional capabilities (Maroufkhani et al., 2022; Priya and Sharma, 2023). This relationship improves users’ perceived value and satisfaction (Song et al., 2022; Lin and Lee, 2024; Song et al., 2024).

In the context of online learning, GAI’s intelligence capacity enables accurate comprehension of users’ inquiries and provision of elaborated responses, thereby enhancing users’ learning experience. Therefore, we hypothesize that:

*H1a*: Intelligence positively influences users’ perceived value.

*H1b*: Intelligence positively influences users’ satisfaction.

Explainable AI (XAI) has been defined as systems designed to provide transparent decision processes and clear explanations, enabling users to understand system capabilities and limitations (Dwivedi et al., 2023). Research has demonstrated that this explainability feature enhances users’ trust and acceptance of recommendation algorithms while simultaneously enabling effective knowledge transfer, thereby increasing the adoptability of AI-generated suggestions (Zhang and Curley, 2018). Fundamentally, explainability refers to an AI system’s capacity to articulate its decision logic in a user-comprehensible format. This capability primarily aims to eliminate the “black box” nature of the AI decision-making process, consequently strengthening users’ confidence in the system (Shin, 2021). Within human-GAI interaction contexts, explainability facilitates rational evaluation of algorithmic outputs by providing decision-making rationales. The statement is echoed by the qualitative investigation. For example, interviewee (P23) mentioned: “*AI can give more detailed content. This thought process is more in line with my idea of recognizing it and learning from it.*” This mechanism significantly shapes users’ attitudes toward GAI (Cheung and Ho, 2025). Furthermore, by bridging a connection between the user’s perception and GAI’s operation, explainability not only deepens understanding of specific responses but also elevates the overall interaction quality. As users progressively acquire more comprehensive explanations of the system through successive cycles of inquiries, it enhances users’ perceived value and satisfaction. Therefore, we hypothesize that:

*H2a*: Explainability positively influences users’ perceived value.

*H2b*: Explainability positively influences users’ satisfaction.

Response time, as a key indicator of AI’s service efficacy, reflects the timeliness of the system in processing users’ requests and providing feedback (DeLone and McLean, 2003; Liu Y. L. et al., 2023). AI, powered by sophisticated machine learning algorithms and natural language processing capabilities, can analyze vast datasets and generate precise responses within milliseconds. This superior responsiveness significantly improves the efficiency of human-computer interaction (Neiroukh et al., 2024). The statement is echoed by the qualitative investigation. For example, interviewee (P14) mentioned: “*If you use MOOC or bilibili, or some other large and well-known platforms, one thing they have in common is that you need to watch videos, which may require some investment in your time cost. But instead, using the generative AI software, it can give you a result in a few seconds. I really like it!*” Research has shown that prolonged response times not only reduce task efficiency but may also convey negative impressions about the system’s predictive capabilities. Users’ prevailing assumption that most prediction tasks should be inherently simple for AI systems (Efendić et al., 2020). Liu Y. L. et al. (2023) also pointed out that response time is one of the determining factors affecting users’ perceived value and satisfaction with an AI service. GAI’s timely response impacts the interaction quality, which subsequently strengthens users’ trust in the GAI (Pham et al., 2024). Especially in online learning scenarios, as the response speed increases, it not only sustains users’ engagement and concentration during GAI interactions but also fosters more positive attitudes toward the technology. Therefore, we hypothesize that:

*H3a*: Response time positively influences users’ perceived value.

*H3b*: Response time positively influences users’ satisfaction.

Integrability means that GAI effectively facilitates the ability to combine information from different sources to respond to users’ problems (Chen et al., 2025). It depends on the task and contextual environment, which also reflects task-related properties (Nelson et al., 2005). Chen et al. (2025) argued that highly interdependent complex tasks are more dependent on the integrated system’s outputs than a task-independent system. GAI can be optimally adapted not only based on existing databases but also combined with historical user interaction data in the content generation process (Chen et al., 2025). Existing studies have shown that optimizing the knowledge acquisition pathways, thereby enhancing learning effectiveness and enabling learners to achieve superior outcomes (Korayim et al., 2025). Furthermore, the integrability facilitates rapid adaptation to environmental changes and effective utilization of pivotal opportunities (Ding, 2021). In online learning contexts, GAI generates systematic and related knowledge according to users’ needs, and this powerful integration capability enables users to flexibly respond to problems, thus fostering a more favorable attitude toward technology. Interviewee (P14) mentioned: “*GAI will organize these words into more complete results and present to me, so it may be more convenient in the ability to integrate information. I think it is better than before we searched the web page.*” Therefore, we hypothesize that:

*H4a*: Integrability positively influences users’ perceived value.

*H4b*: Integrability positively influences users’ satisfaction.

Accuracy represents that the system provides up-to-date and relevant information to the users’ intended goals (Chung et al., 2020). It is one of the important foundations for users to use smart service products (Cheng and Jiang, 2022). This viewpoint is echoed by P9, who mentioned: “*There are some official data or real-time information, and I do not 100% believe the answers it gives me.*” During the interaction process, it is often necessary to donate considerable cognitive efforts to evaluate the precision and relevance of GAI-generated content (Chen et al., 2023c). When users confirm that AI recommendations sufficiently address their requirements, this validation triggers a “cognitive resonance” phenomenon—the perception that the system genuinely comprehends their underlying needs—thereby substantially increasing recommendation acceptance (Li et al., 2021). Accuracy not only makes users feel that their needs are fully valued but, more importantly, provides effective solutions (Yuan et al., 2022). This positive experience strengthens users’ recognition of AI technology capabilities (Gursoy et al., 2019), and plays an important role in users’ satisfaction (Walle et al., 2023). In the context of online learning, accuracy is of equal importance. GAI should ensure the solutions and methods are correct and feasible to establish perceived value, fostering positive user attitudes (Chen et al., 2023b). Therefore, we hypothesize that:

*H5a*: Accuracy positively influences users’ perceived value.

*H5b*: Accuracy positively influences users’ satisfaction.

Source credibility defined as individuals’ perception of sources as trustworthy and expertized (Hovland and Weiss, 1951). Camilleri (2024) stated that users often rely on pre-existing perceptions about information sources rather than objectively assessing content quality. It is a prerequisite for users to assess information’s usefulness (Camilleri and Filieri, 2023). Compared to non-expert sources, information disseminated by experts is usually perceived higher reliability and credibility (Ismagilova et al., 2020). Users will be more inclined to accept the advice and knowledge provided by professional and authoritative sources (Hovland and Weiss, 1951; Wang and Scheinbaum, 2018). In the context of online learning, users expect the source reliability from GAI. This viewpoint is echoed by P21, who mentioned: “*If the results of GAI are different from those I searched in Baidu, I may not be able to judge which information is true.*” Only when they confirm information originates from credible and authoritative sources do they feel confident acquiring knowledge through GAI interactions. This credibility reinforces users’ perception of information utility (Camilleri and Kozak, 2022), as well as contributing to positive attitudes toward GAI. Therefore, we hypothesize that:

*H6a*: Source credibility positively influences users’ perceived value.

*H6b*: Source credibility positively influences users’ satisfaction.

Personalization provides customized services to users based on their needs, preferences, and intent (Ameen et al., 2021). Personalization in AI-based services has been defined as the service capability to provide specialized services based on a user’s personal information and contextual usage (Liu and Tao, 2022; Kim and Hur, 2024). Highly personalized AI not only formulates precise inquiries to identify individual needs but also simulates users’ decision-making processes (Kim and Hur, 2024). Therefore, it generates customized content that better meets the users’ expectations. Pappas et al. (2017) demonstrated that users are more inclined to higher relevant information. Aw et al. (2024) further noted that higher levels of personalization in mobile AR shopping apps can create a stronger sense of realism and coherence between virtual and real dimensions, which can lead to a significant increase in users’ immersion. In online learning scenarios, when GAI provides personalized answers to users’ specific questions, it optimizes satisfaction (Li and Zhang, 2023), and effectively enhances users’ perceived value. Such personalized learning support better matches the educational content with the learner’s knowledge level and cognitive style, thus creating a more efficient and enjoyable learning experience (Baillifard et al., 2025). Interviewee (P6) mentioned: “*Because many of my questions will be very professional, I would prefer to have such a personalized and professional AI.*” Therefore, we hypothesize that:

*H7a*: Personalization positively influences users’ perceived value.

*H7b*: Personalization positively influences users’ satisfaction.

Emotional support, as a crucial dimension of social support, fulfills individuals’ psychological needs by conveying empathy, emotional validation, and encouragement (Meng and Dai, 2021). Existing research suggested that human-provided emotional support effectively enhanced individuals’ role meaning, thereby increasing well-being (Pai, 2023), as well as significantly improving service evaluations (Menon and Dubé, 2007) and effectively alleviating psychological stress (Meng and Dai, 2021). GAI can simulate human emotional communication, and are gradually taking on the role of emotional support (Gelbrich et al., 2021). This viewpoint is echoed by interviewee P16, who mentioned: “*I think GAI is completely different from human’s feedback. Maybe humans will tell you that the employment environment is not very good for finding a job, and the situation is not very optimistic now. But GAI is relatively neutral. It may just give me some vitality.*” Lee et al. (2022) pointed out that AI chatbots equipped with emotional intelligence dialogue systems can provide emotional understanding and encouragement to users. This interaction facilitates deeper communication and builds emotional connections. In our interviews, participants also reported that when they experienced academic stress and confided in GAI, they received both emotional consolation and personalized academic guidance. In online learning scenarios, GAI’s emotional support creates a friend-like interactive experience, and this humanized interaction significantly enhances users’ perceived value and satisfaction. Therefore, we hypothesize that:

*H8a*: Emotional support positively influences users’ perceived value.

*H8b*: Emotional support positively influences users’ satisfaction.

Perceived value represents users’ overall evaluation of a product or service’s usefulness (Chen et al., 2023b). Within human-GAI interaction contexts, this construct is users’ assessment of GAI’s functionality, such as its 24/7 availability, problem-solving efficiency, and generation of content (Carvalho and Ivanov, 2024; Xu et al., 2024). As Lee et al. (2007) stated that perceived value is more significant in increasing satisfaction than service quality. Perceived value is different in scenarios, such as technology, service delivery, and tangible commitments (De Kervenoael et al., 2020). In AI service applications, robots can effectively balance temporal efficiency, economic considerations, and user experience, which directly influence users’ perceived value (De Kervenoael et al., 2020). Interviewee (P10) mentioned: “*I may still need to spend time identifying what is right and wrong. But it will take less time than Baidu. This effectively offsets the time spent identifying the answer and is important for my experience of using it afterwards.*” Infusion use is an active, repetitive, and long-term deep use behavior of GAI (Hu et al., 2024), which represents a more sustained engagement than continuous use behavior. In new technology research, perceived value is a critical factor that influences users’ long-term use behavior (Lavado-Nalvaiz et al., 2022; Maroufkhani et al., 2022). Therefore, we hypothesize that:

*H9*: Perceived value positively influences users’ infusion use.

*H11a*: Perceived value mediates the relationship between intelligence and infusion use.

*H11b*: Perceived value mediates the relationship between explainability and infusion use.

*H11c*: Perceived value mediates the relationship between response time and infusion use.

*H11d*: Perceived value mediates the relationship between integrability and infusion use.

*H11e*: Perceived value mediates the relationship between accuracy and infusion use.

*H11f*: Perceived value mediates the relationship between source credibility and infusion use.

*H11g*: Perceived value mediates the relationship between personalization and infusion use.

*H11h*: Perceived value mediates the relationship between emotional support and infusion use.

Satisfaction is an indicator of service quality assessment, quantifying the variance between users’ actual service experience and their expectations (Xie et al., 2023). This concept encompasses not only the immediate usage pleasure but also a comprehensive assessment process that involves the comparison of users’ past experiences with their current expectations (Poushneh et al., 2024). When using AI services, satisfaction is one of the main factors that impact users’ subsequent behavior (Jiang et al., 2022; Chen et al., 2023b; Xie et al., 2024). This viewpoint is echoed by interviewee P25, who mentioned: “*GAI, like Doubao, is helpful, because it will make me more and more fluent, and then the expression will become more and more natural, and the response will get better and better in all aspects. That’s why I’ve been sticking with it for oral speaking.*”Specifically, when the GAI provides information that aligns with user requirements, this positive experience fosters favorable beliefs, ultimately promoting their sustained behavior (i.e., infusion use) (Ku and Chen, 2024). Therefore, we hypothesize that:

*H10*: Satisfaction positively influences users’ infusion use.

*H12a*: Satisfaction mediates the relationship between intelligence and infusion use.

*H12b*: Satisfaction mediates the relationship between explainability and infusion use.

*H12c*: Satisfaction value mediates the relationship between response time and infusion use.

*H12d*: Satisfaction mediates the relationship between integrability and infusion use.

*H12e*: Satisfaction mediates the relationship between accuracy and infusion use.

*H12f*: Satisfaction mediates the relationship between source credibility and infusion use.

*H12g*: Satisfaction mediates the relationship between personalization and infusion use.

*H12h*: Satisfaction mediates the relationship between emotional support and infusion use.

**Figure 2:**
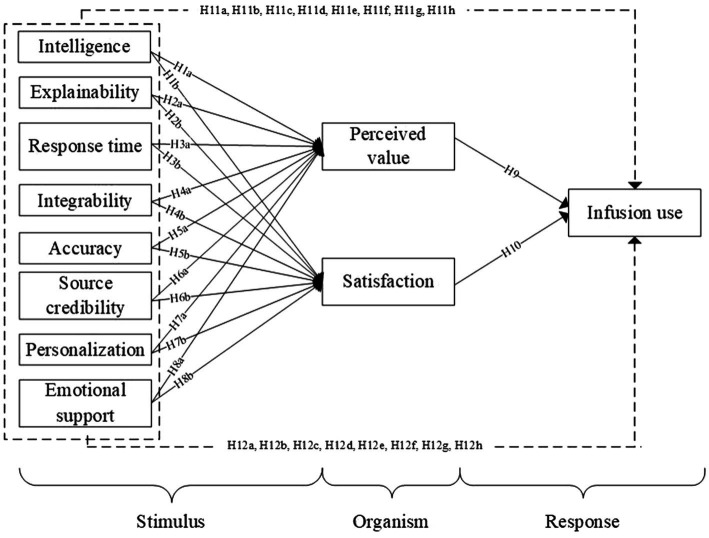
The proposed research model.

Figure 2 describes the theoretical model.

### 4.2 Questionnaire design

This study collected data through a questionnaire comprising three main sections. The first section outlines the research purpose, defines GAI, and presents two examples of its application in online learning scenarios. The second section measures eight antecedent factors, two mediators, and the outcome variable in the theoretical model. The third section captures participants’ demographic information, including gender, age, education level, profession, frequency and year of GAI usage in learning.

To ensure the reliability and validity of the questionnaire, the measurement items in this study were adapted from established scales in the literature, with appropriate modifications to the context of GAI in online learning scenarios. Among them, intelligence, response time, and explainability use the scales developed by scholars such as Mehmood et al. (2024), Darban (2024), and Liu Y. L. et al. (2023), respectively. The scale of integration, accuracy, and source credibility mainly refers to the study of Chen et al. (2025), Zhou and Wu (2024), Yuan et al. (2022), and Wilson and Baack (2023). Personalization and emotional support are mainly based on the scales developed by Chen et al. (2023a), Zhu et al. (2023), and Zhang et al. (2018). Perceived value is adapted from De Kervenoael et al. (2020) and Chen et al. (2023b). Satisfaction is based on Xu et al. (2023). Finally, infusion use is adapted from Hu et al. (2024). All questionnaire research data were collected by a seven-point Likert scale (1 = strongly disagree, 7 = strongly agree). Detailed measurement items are provided in Appendix B Table B1.

Before the formal survey, this study conducted a pilot test involving fifty participants and consulted experts to refine the wording and structure of the questionnaire items based on the participants’ feedback. The pilot test results indicated that the scale demonstrated strong reliability and validity.

### 4.3 Data collection

Questionnaire data for this study were collected via Sojump,^3^ a widely used online survey platform in China with 260 million registered users, similar to Amazon MTurk (Wu et al., 2024). This platform has also been widely used in previous related studies (Ding et al., 2023; Del Ponte et al., 2024; Javed et al., 2024). Before the questionnaire, participants were provided with a brief introduction to GAI and online learning, along with two screenshots demonstrating the use of GAI in online learning contexts. After the questionnaire is completed, each participant will receive 3 RMB (about 0.413 $) as a reward. The questionnaire was distributed and collected in February 2025, yielding 386 participants who joined our study.

We obtained data through random sampling, but to ensure the quality of the data, this study implemented three data screening criteria. First, participants were required to have prior experience using GAI for learning purposes. The question, “Have you ever used GAI to assist in learning?” was set to exclude participants without experience (*N* = 17). Second, two attention tests were conducted to exclude the sample who failed to answer correctly (*N* = 23). Additionally, the reverse questions were included for the third item of explainability and the fourth item of accuracy to drop samples with inconsistent responses (*N* = 19). In sum, we included 327 participants in our data analysis. Following Chin’s (1998) guideline for PLS-SEM, we ensured the sample size exceeded both: (1) 10 times the number of items in the largest construct; (2) 10 times the number of independent variables. Second, we used G*Power 3.1.9 software to calculate the sample size. With a significance level α= 0.05, Power(1−β)= 0.95, and effect size = 0.15, the minimum sample size was 160. Our sample sizes satisfy these requirements. Among the participants, 160 were female (48.93%), with the majority aged 19–24 (41.59%). The data on year of usage indicated that users with 1–2 years of experience constitute the majority (51.07%). The demographics of the final sample are presented in Table 2.

**Table 2:** Sample demographics.

Item	Indicators	Number	Percentage (%)
Gender	Male	167	51.07
	Female	160	48.93
Age	19-24	136	41.59
	25-33	89	27.22
	34-44	75	22.94
	>=45	27	8.26
Education level	High school or below	26	7.95
	College	76	23.24
	Undergraduate	185	56.57
	Postgraduate and higher	40	12.23
Profession	Student	199	60.86
	Employees of Government or institutions	37	11.31
	Company Employee	52	15.90
	Self-employed	17	5.20
	Other	22	6.73
Frequency of learning with GAI	1-2 times per week	35	10.70
	3-4 times per week	95	29.05
	5-6 times per week	119	36.39
	Everyday	78	23.85
Years of learning with GAI	Within 1 year	42	12.84
	1-2 year	167	51.07
	More than 2 years	118	36.09

### 4.4 Data analysis

We used Partial Least Square (PLS) to test our theoretical model. PLS-SEM has no strict requirements on sample size and quantity. In addition, it has strong predictive and interpretative ability (Hair et al., 2011), which is suitable for exploratory theoretical construction. The influence mechanism of this study first built a model of GAI’s infusion use in online learning scenarios, which belongs to exploratory research and is suitable for the PLS-SEM method. The data were analyzed using SmartPLS 4.0. We followed the two-step approach in examining the measurement and structural models.

#### 4.4.1 Measurement model

To ensure the reliability and validity of the questionnaire, we assessed its convergent and discriminant validity and reliability (MacKenzie et al., 2011), with the results detailed in Table 3. Specifically, reliability was assessed by Cronbach’s Alpha and Composite reliability (CR) for all variables. The results indicate that the Cronbach’s Alpha values of all variables range from 0.820 to 0.892, and the CR values range from 0.893 to 0.921, with all coefficients exceeding the threshold of 0.7, which indicates that the questionnaire has strong internal consistency. Additionally, the average variance extracted (AVE) values for each construct ranged between 0.675 and 0.752, all exceeding 0.5, providing evidence of convergent validity.

**Table 3:** Assessment of reliability and convergent validity.

Constructs	Items	Loading	Cronbach’s alpha	CR	AVE
Intelligence	INTEL1	0.842	0.897	0.924	0.708
	INTEL2	0.858			
	INTEL3	0.833			
	INTEL4	0.850			
	INTEL5	0.825			
Explainability	EXP1	0.862	0.920	0.940	0.757
	EXP2	0.874			
	EXP3	0.885			
	EXP4	0.861			
	EXP5	0.867			
Response time	RT1	0.884	0.840	0.904	0.757
	RT2	0.871			
	RT3	0.856			
Integrability	INTEG1	0.850	0.834	0.900	0.750
	INTEG2	0.892			
	INTEG3	0.856			
Accuracy	ACC1	0.837	0.885	0.920	0.743
	ACC2	0.868			
	ACC3	0.885			
	ACC4	0.857			
Source credibility	SC1	0.845	0.913	0.935	0.741
	SC2	0.877			
	SC3	0.876			
	SC4	0.848			
	SC5	0.859			
Personalization	PER1	0.838	0.906	0.930	0.727
	PER2	0.845			
	PER3	0.863			
	PER4	0.876			
	PER5	0.841			
Emotional support	ES1	0.835	0.876	0.915	0.729
	ES2	0.869			
	ES3	0.863			
	ES4	0.849			
Perceived value	PV1	0.867	0.902	0.931	0.772
	PV2	0.890			
	PV3	0.883			
	PV4	0.876			
Satisfaction	SAT1	0.903	0.895	0.934	0.826
	SAT2	0.911			
	SAT3	0.908			
Infusion use	IU1	0.850	0.878	0.916	0.732
	IU2	0.860			
	IU3	0.858			
	IU4	0.856			

Discriminant validity was assessed using the Fornell-Larcker criterion and Heterotrait-monotrait ratio (HTMT). Table 4 shows that the square root of the AVE values of all constructs was higher than the inter-construct correlations (MacKenzie et al., 2011), demonstrating good discriminant validity. Furthermore, the HTMT values among the constructs in Table 5 are below the critical value of 0.85.

**Table 4:** Discriminant validity using (Fornell-Larcker method).

	INTEL	EXP	RT	INTEG	ACC	SC	PER	ES	PV	SAT	IU
INTEL	**0.842**										
EXP	0.259	**0.870**									
RT	0.365	0.448	**0.870**								
INTEG	0.274	0.251	0.342	**0.866**							
ACC	0.297	0.249	0.291	0.433	**0.862**						
SC	0.279	0.369	0.379	0.500	0.461	**0.861**					
PER	0.259	0.451	0.373	0.393	0.369	0.561	**0.853**				
ES	0.201	0.326	0.291	0.470	0.404	0.633	0.576	**0.854**			
PV	0.363	0.498	0.469	0.526	0.491	0.651	0.605	0.663	**0.879**		
SAT	0.410	0.514	0.527	0.545	0.536	0.640	0.588	0.608	0.745	**0.909**	
IU	0.423	0.524	0.510	0.429	0.418	0.543	0.550	0.488	0.695	0.721	**0.856**

INTEL refers to intelligence, EXP refers to explainability, RT refers to response time, INTEG refers to integrability, ACC refers to accuracy, SC refers to source credibility, Per refers to personalization, ES refers to emotional support. PV refers to perceived value. SAT refers to satisfaction. IU refers to infusion use. Diagonal elements are the square root of the average extracted (AVE).

**Table 5:** Discriminant validity using (HTMT method).

	INTEL	EXP	RT	INTEG	ACC	SC	PER	ES	PV	SAT	IU
INTEL	–										
EXP	0.286	–									
RT	0.421	0.509	–								
INTEG	0.313	0.285	0.404	–							
ACC	0.332	0.273	0.337	0.500	–						
SC	0.308	0.403	0.433	0.570	0.513	–					
PER	0.287	0.494	0.427	0.448	0.410	0.617	–				
ES	0.226	0.364	0.339	0.545	0.458	0.708	0.648	–			
PV	0.403	0.546	0.538	0.603	0.548	0.718	0.669	0.745	–		
SAT	0.457	0.565	0.608	0.627	0.599	0.707	0.652	0.685	0.829	–	
IU	0.477	0.582	0.594	0.498	0.471	0.606	0.616	0.556	0.781	0.813	–

INTEL refers to intelligence, EXP refers to explainability, RT refers to response time, INTEG refers to integrability, ACC refers to accuracy, SC refers to source credibility, PER refers to personalization, ES refers to emotional support. PV refers to perceived value. SAT refers to satisfaction. IU refers to infusion use.

#### 4.4.2 Common method bias

Since the sample data were collected from a single source, with participants answering all questions simultaneously, this may lead to common method bias (CMB) among them. To mitigate the potential impact of CMB, this study adopted several control measures based on established studies: (1) informing participants that the survey was anonymous and that there were no right or wrong answers; (2) incorporating reverse items and attention test questions; and (3) balancing the order of questionnaire items. Additionally, we used Harman’s single-factor test for CMB. The results of a principal component analysis indicated that a single factor explains 38.50% of the variance in the data, which is below the recommended threshold of 40% (Podsakoff et al., 2003). Furthermore, this study employed the method proposed by Liang et al. (2007), and the results are presented in Appendix B Table B2. The average substantive explained factor loading (0.746) was larger than the average method factor loading (0.002), yielding a ratio of 493:1. Both test results imply that the CMB may not be a concern.

#### 4.4.3 Structure model

To access the structural model, this study evaluated the variance explained ($R^{2}$), effect size ($f^{2}$), and Stone-Geisser’s ($Q^{2}$) of variables. The model explained a portion of the variance, with a coefficient of determination ($R^{2}$) of 0.648 for perceived value, 0.661 for satisfaction, and 0.576 for infusion use as a dependent variable, indicating a good level of predictive power (Hair et al., 2019). Effect size analysis ($f^{2}$) showed all values ranging from 0.016 to 0.218, indicating low to medium impacts across constructs (Chin, 1998). Additionally, all the $Q^{2}$ values exceeded the threshold of zero, confirming the model’s relevance regarding all endogenous variables (Hair et al., 2019).

A bootstrapping procedure was chosen to measure the significance of the path coefficient, standard error, and *t*-statistics (Table 6). (1) Perceived value. The results of the path analysis suggested that perceived value was positively influenced by intelligence (*β* = 0.084, *p* < 0.05), explainability (*β* = 0.162, *p* < 0.001), response time (*β* = 0.101, *p* < 0.05), integrability (*β* = 0.111, *p* < 0.05), accuracy (*β* = 0.106, *p* < 0.01), source credibility (*β* = 0.172, *p* < 0.01), personalization (*β* = 0.129, *p* < 0.05) and emotional support (*β* = 0.285, *p* < 0.001). These results supported H1a, H2a, H3a, H4a, H5a, H6a, H7a and H8a. (2) Satisfaction. The results of the path analysis suggested that satisfaction was positively associated with intelligence (*β* = 0.111, *p* < 0.01), explainability (*β* = 0.167, *p* < 0.001), response time (*β* = 0.161, *p* < 0.001), integrability (*β* = 0.134, *p* < 0.01), accuracy (*β* = 0.165, *p* < 0.01), source credibility (*β* = 0.162, *p* < 0.01), personalization (*β* = 0.111, *p* < 0.05) and emotional support (*β* = 0.188, *p* < 0.001). These results supported H1b, H2b, H3b, H4b, H5b, H6b, H7b and H8b.

**Table 6:** Direct effects test.

Hypothesis	Path	Path coefficients	*t*-statistics	*p* values	Results
H1a	INTEL - > PV	0.084	2.542	0.011	Supported
H1b	INTEL - > SAT	0.111	3.066	0.002	Supported
H2a	EXP - > PV	0.162	4.007	0.000	Supported
H2b	EXP - > SAT	0.167	4.420	0.000	Supported
H3a	RT - > PV	0.101	2.422	0.015	Supported
H3b	RT - > SAT	0.161	3.772	0.000	Supported
H4a	INTEG - > PV	0.111	2.324	0.020	Supported
H4b	INTEG - > SAT	0.134	3.125	0.002	Supported
H5a	ACC - > PV	0.106	2.665	0.008	Supported
H5b	ACC - > SAT	0.165	3.339	0.001	Supported
H6a	SC - > PV	0.172	2.711	0.007	Supported
H6b	SC - > SAT	0.162	2.803	0.005	Supported
H7a	PER - > PV	0.129	2.229	0.026	Supported
H7b	PER - > SAT	0.111	2.505	0.012	Supported
H8a	ES - > PV	0.285	4.353	0.000	Supported
H8b	ES - > SAT	0.188	4.007	0.000	Supported
H9	PV - > IU	0.356	4.973	0.000	Supported
H10	SAT - > IU	0.456	6.451	0.000	Supported

INTEL refers to intelligence, EXP refers to explainability, RT refers to response time, INTEG refers to integrability, ACC refers to accuracy, SC refers to source credibility, PER refers to personalization, ES refers to emotional support. PV refers to perceived value. SAT refers to satisfaction. IU refers to infusion use. The number of Bootstrap samples = 5,000.

**Figure 3:**
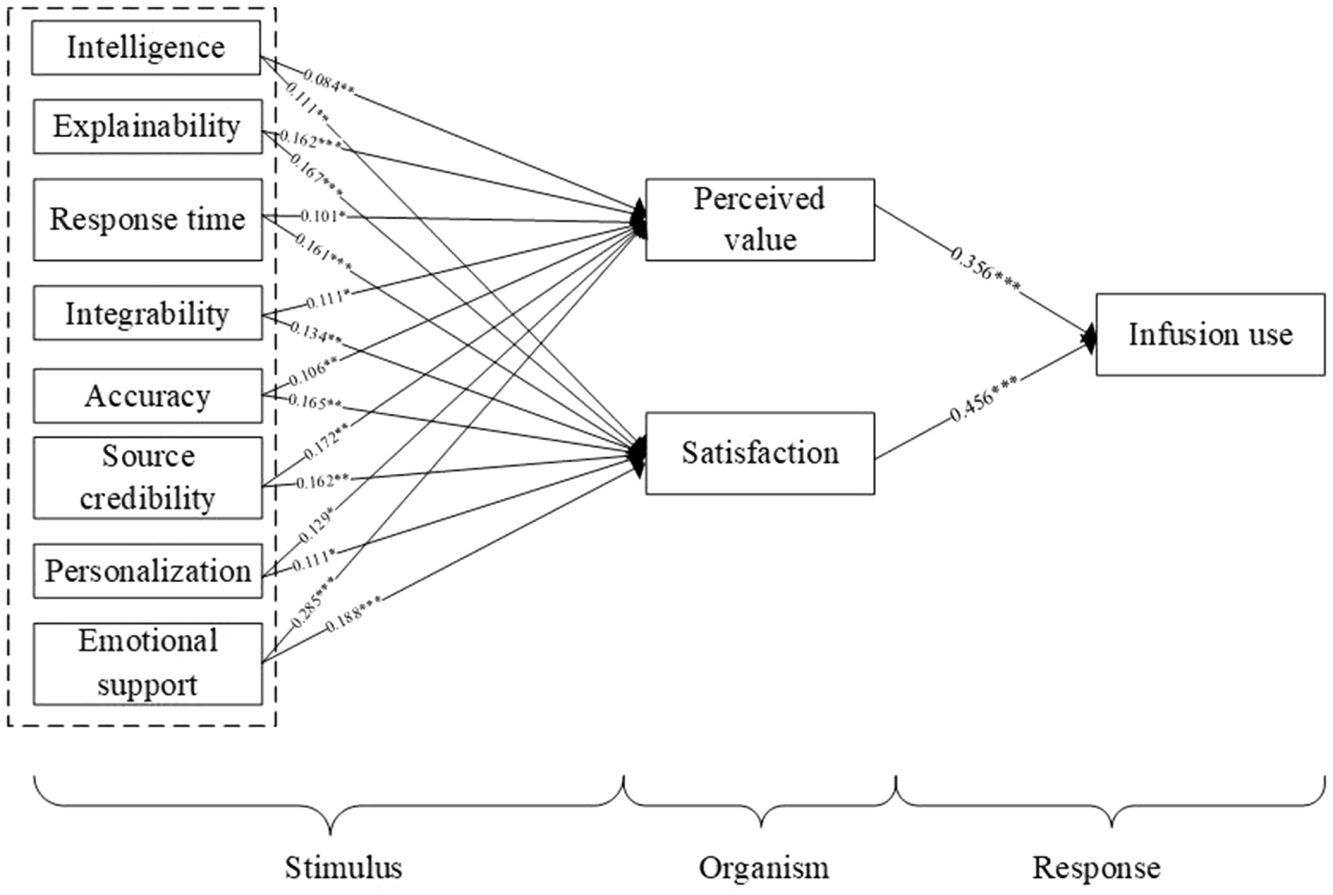
Direct effects test.

Perceived value was found to be positively associated with infusion use (*β* = 0.356, *p* < 0.001), thus supporting H9. Satisfaction was positively influenced by infusion use (*β* = 0.456, *p* < 0.001), which supported H10. As shown in Figure 3, the hypothesis proposed in this study is supported.

Furthermore, we conducted mediation tests on the effects of perceived value and satisfaction. Specifically, we utilized the bootstrapping method with 5,000 repetitions to construct confidence intervals (CIs) (Edwards and Lambert, 2007; Hayes, 2009). Table 7 presents the bootstrapping results along with the corresponding 95% CIs. It shows that perceived value partially or fully mediates the relationship between intelligence, explainability, response time, integrability, accuracy, source credibility, personalization, emotional support, and infusion use. These results supported H11a, H11b, H11c, H11d, H11e, H11f, H11g, H11h. At the same time, satisfaction partially or fully mediates the relationship between intelligence, explainability, response time, integrability, accuracy, source credibility, personalization, emotional support, and infusion use. These results supported H12a, H12b, H12c, H12d, H12e, H12f, H12g, H12h.

**Table 7:** Indirect and mediating effects test.

Relationship			Direct effect		95% Bias-Corrected CI			Results
IV	M	DV	Effect	*p* values	Effect	LLCI	ULCI	
INTEL	PV	IU	0.203	0.000	0.202	0.119	0.286	Partial
EXP	PV	IU	0.224	0.000	0.271	0.209	0.341	Partial
RT	PV	IU	0.239	0.000	0.275	0.213	0.344	Partial
INTEG	PV	IU	0.087	0.066	0.342	0.274	0.414	Full
ACC	PV	IU	0.099	0.028	0.313	0.242	0.385	Partial
SC	PV	IU	0.149	0.003	0.367	0.298	0.437	Partial
PER	PV	IU	0.200	0.000	0.342	0.266	0.420	Partial
ES	PV	IU	0.048	0.363	0.433	0.349	0.526	Full
INTEL	SAT	IU	0.159	0.002	0.276	0.204	0.351	Partial
EXP	SAT	IU	0.200	0.000	0.298	0.235	0.363	Partial
RT	SAT	IU	0.182	0.001	0.332	0.258	0.412	Partial
INTEG	SAT	IU	0.052	0.259	0.376	0.303	0.457	Full
ACC	SAT	IU	0.456	0.311	0.367	0.301	0.437	Full
SC	SAT	IU	0.133	0.005	0.382	0.300	0.471	Partial
PER	SAT	IU	0.191	0.000	0.351	0.277	0.430	Partial
ES	SAT	IU	0.080	0.093	0.401	0.321	0.481	Full

INTEL refers to intelligence, EXP refers to explainability, RT refers to response time, INTEG refers to integrability, ACC refers to accuracy, SC refers to source credibility, PER refers to personalization, ES refers to emotional support. PV refers to perceived value. SAT refers to satisfaction. IU refers to infusion use. The number of Bootstrap samples = 5,000.

#### 4.4.4 Fuzzy-set qualitative comparative analysis (fsQCA)

**Figure 4:**
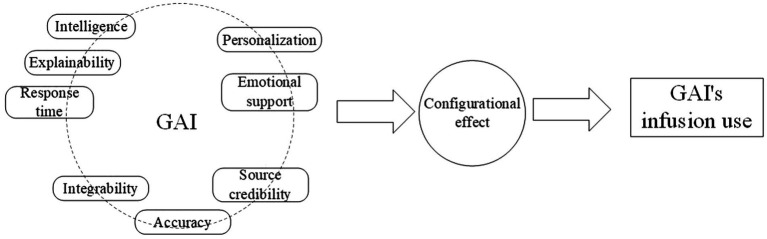
Configurational model of users’ infusion use of GAI.

The theoretical foundation of SEM is based on the principle of correlational causation. This implies that variations in independent variables systematically influence dependent variable values. However, this assumption’s reliability may be limited due to the fundamentally asymmetric nature of most real-world relationships (Chakraborty et al., 2024). fsQCA tackles these concerns and integrates the strengths of both qualitative and quantitative research methods, accommodating sample sizes ranging from very small to very large (Lin et al., 2024). It is particularly well-suited for examining complex relationships among multiple factors in social phenomena. This study employs fsQCA for two primary reasons. First, it can better explore the causal complexity (Wang et al., 2024). Given that GAI infusion use is driven by multiple factors, traditional quantitative methods, which often isolate the individual effects of each factor, are less suitable (Hu et al., 2024). Second, fsQCA is based on Boolean algebra rather than regression analysis, enabling any situation to be described as a combination of causal conditions and their outcomes. Through logical and non-statistical procedures, fsQCA can establish logical links between combinations of causal conditions and outcomes (Lin et al., 2024), thereby providing deeper insights into the mechanisms that shape users’ infusion use. Figure 4 shows the configuration of antecedents and outcome conditions.

According to Pappas and Woodside (2021), the fsQCA method has three stages: (1) data calibration; (2) necessary conditions; (3) configuration analysis. We used the fsQCA 3.0 software to analyze the 327 samples. As recommended of fsQCA studies, variable calibration was conducted before the analysis of necessary conditions. Specifically, three calibration anchors — the full membership, the crossover point, and the full non-membership — were defined. According to previous research, 95, 50, and 5% of each construct were used to set full membership, crossover point, and full non-membership, respectively (Lalicic and Weismayer, 2021). This process transformed the questionnaire data into continuous membership scores ranging from 0 to 1 (Zhou and Wu, 2024). Additionally, following Ragin (2006), a value of 0.001 was added to the calibrated values to avoid excessive 0.5 that would result in data exclusion during truth table construction.

We further used the fsQCA 3.0 software to strengthen the accuracy of necessary condition analysis. If the consistency threshold is greater than 0.9, the antecedent condition is a necessary condition. The results are presented in Table 8. We can see that the maximum consistency of the antecedent conditions that influence infusion use is 0.841. It indicates that all the antecedent conditions are not necessary conditions. Therefore, it is necessary to understand users’ infusion use through configurational analysis.

**Table 8:** Analysis of necessary conditions of fsQCA method.

Antecedents	Infusion use	
	Consistency	Coverage
Intelligence	0.725	0.754
~Intelligence	0.481	0.442
Response time	0.785	0.767
~Response time	0.421	0.411
Explainability	0.768	0.784
~Explainability	0.420	0.392
Integrability	0.829	0.742
~Integrability	0.372	0.399
Accuracy	0.804	0.756
~Accuracy	0.392	0.397
Source credibility	0.841	0.786
~Source credibility	0.348	0.356
Personalization	0.820	0.786
~Personalization	0.363	0.361
Emotional support	0.841	0.786
~Emotional support	0.354	0.362

“~” means NOT in logical operators.

We constructed a truth table for all logically possible antecedent configurations. Following Pappas and Woodside (2021), the case frequency threshold was set at 3 when the sample size exceeded 150. In this study, the consistency threshold was set at 0.85, and PRI consistency thresholds below 0.75 were labeled as 0. Variables that appear in both intermediate and parsimonious solutions are considered as core conditions, while those appearing only in intermediate solutions are identified as peripheral conditions (Fiss, 2011). The results of the configurations are shown in Table 9. The large filled circles (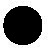) represent the presence of core conditions, while the small filled circles (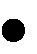) indicate the presence of peripheral conditions. Conversely, denote the absence of core conditions, and the small cross-out circles (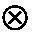) signify the absence of peripheral conditions. Blank indicates the condition is present or absent. As shown in the table, six configurations explain users’ infusion use, with an overall solution consistency of 0.655 and a coverage of 0.957. Both indicators exceed the recommendation, confirming the reliability of the results (Li F. et al., 2024). It shows that the six configurations are highly explanatory for users’ infusion use. Among these, S4a and S4b constitute a second-order equivalent configuration, as their core conditions are identical.

**Table 9:** Sufficient configurations for infusion use.

Antecedent conditions	Infusion use					
	S1	S2	S3	S4a	S4b	S5
Intelligence	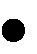			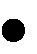		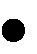
Response time	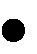	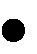	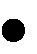			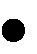
Explainability		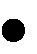	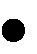		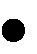	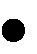
Integrability	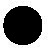	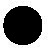		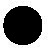	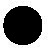	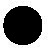
Accuracy	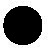	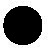	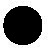	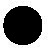	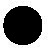	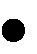
Source credibility	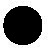		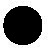	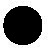	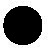	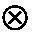
Personalization		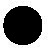	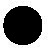	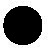	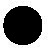	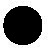
Emotional support	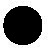	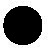	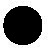	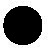	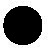	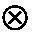
Raw coverage	0.394	0.405	0.397	0.404	0.416	0.154
Unique coverage	0.042	0.049	0.040	0.021	0.027	0.024
Consistency	0.965	0.967	0.962	0.966	0.960	0.969
Overall solution coverage	0.655					
Overall solution consistency	0.957					

S1 indicates that when GAI possesses integrability, accuracy, source credibility, and emotional support as core conditions, along with intelligence and response time as peripheral conditions, users are more likely to infusion use it as a tool in everyday learning. S2 demonstrates that GAI with integrability, accuracy, personalization, and emotional support as core conditions complemented by response time and explainability as peripheral conditions, enhances users’ infusion use. S3 shows that GAI, with accuracy, source credibility, personalization, and emotional support as core conditions, and response time and explainability as peripheral conditions, leads to high infusion use. S4a shows that when GAI has integrability, accuracy, source credibility, personalization, and emotional support as core conditions, with intelligence as a peripheral condition, it significantly strengthens users’ infusion use. In addition, S4b exhibits the highest raw coverage and represents the core configuration for infusion use. It shares the same core conditions as S4a but includes explainability as a peripheral condition, both positively influencing users’ infusion use. S5 illustrates that GAI with integrability and personalization as core conditions exist, combined with intelligence, response time, explainability, and accuracy as peripheral conditions, facilitates users’ infusion use even in the absence of source credibility and emotional support.

We tested the sensitivity of the solutions to both the sample and the calibration. First, the consistency threshold was reduced from 0.85 to 0.8, with all other parameters unchanged, resulting in configurations identical to the original. Second, the PRI consistency is increased from 0.75 to 0.8, and the rest is unchanged. Compared to the PRI of 0.75, only S5 is eliminated, and the coverage is reduced from 0.655 to 0.631. It can be seen that the results prove to be predominantly robust.

## 5 Discussion

With the widespread application of GAI, individuals are increasingly shifting from traditional online learning platforms (e.g., MOOCs) to GAI-assisted problem-solving. This transition not only transforms users’ learning habits but also raises questions regarding GAI’s impact on user behavior and psychological processes (Kim et al., 2021; Ahmad et al., 2023; Germinder and Capizzo, 2024). To address these questions, this study employed a mixed-methods approach. In the first stage, we conducted 26 semi-structured interviews to identify factors influencing users’ infusion use of GAI in online learning contexts. The second stage comprises a quantitative study that employs 327 participants to validate the proposed research model. Finally, fsQCA was applied to examine the configurational effects among these factors, revealing the distinct pathways that lead to users’ infusion use of GAI.

Guided by “top-down” framework analysis and “bottom-up” grounded theory, stage 1 identifies eight critical factors influencing users’ infusion use of GAI: intelligence, explainability, response time, integrability, accuracy, source credibility, personalization, and emotional support. These factors collectively influence users in establishing deep, long-term engagement with GAI, enabling integration into their daily lives (Jones et al., 2002). Empirical evidence confirms that these eight factors influence users’ infusion use through parallel mediation of perceived value and satisfaction. These findings make up the framework for understanding GAI infusion use in online learning contexts. Beyond previous studies identified influencing factors such as response time (Baabdullah, 2024), accuracy (Zhou and Wu, 2024), and source credibility (Chakraborty et al., 2024) in usage intention, users also place equal importance on other dimensions, such as emotional support and personalization. This distinction highlights GAI’s unique position as an emerging learning tool that combines the accessibility of traditional online education with adaptive capabilities that enhance its responsiveness to individual learning needs.

Our research extends beyond previous studies that examined single or combined factors such as hedonic motivation, habit, perceived usefulness, perceived risk, and perceived responsiveness (Sanusi et al., 2024; Zhao et al., 2024; Kim et al., 2025). This study employs a mixed-methods approach to systematically identify and validate multidimensional factors and process a more comprehensive theoretical framework. Furthermore, our findings regarding response time diverge from Gnewuch et al. (2022). That study concluded that delayed responses align better with human conversational rhythms, effectively stimulating social reactions. However, we found that rapid responses significantly enhance perceived value and satisfaction in online learning context. This discrepancy may reflect users’ expectations that modern technology can maintain both speed and quality (Neiroukh et al., 2024), leading them to associate faster responses with more enjoyable experiences (Yang, 2023).

Finally, through fsQCA analysis, we demonstrate that GAI infusion use is driven by synergistic combinations of multiple factors. While most studies have examined users’ attitudes from a single-factor perspective (Chakraborty et al., 2024; Wang, 2025), our configurational analysis reveals six distinct pathways to infusion use. S4a and S4b form a second-order equivalence configuration. The eight factors function as core or peripheral conditions. Among these, integrability, accuracy, source credibility, personalization, and emotional support emerge as core conditions, with explainability as a peripheral condition, representing the most generalized configurations. Importantly, no single factor constitutes a necessary condition for infusion use.

## 6 Implications and conclusions

### 6.1 Theoretical implications

First, this study systematically proposes a theoretical framework that GAI’s characteristics influence users’ infusion use in online learning scenarios. Previous studies have investigated intelligence and explainability in promoting positive GAI usage (Al-Emran et al., 2024; Darban, 2024; Theresiawati et al., 2025). However, these studies often focus on a single or limited characteristic. Our work comprehensively identifies eight key GAI characteristics influencing users’ infusion use—intelligence, explainability, response time, integrability, accuracy, source credibility, personalization, and emotional support. This integrated theoretical framework not only enriches our understanding of GAI attributes but also provides a systematic theoretical foundation for subsequent research.

Second, this study extends theoretical understanding of perceived value and satisfaction in online learning contexts. Previous research has examined either attitudes (i.e., satisfaction) or cognition (i.e., perceived value) as independent mediators influencing GAI usage in online learning (Chan and Zhou, 2023; Kim et al., 2025). However, the synergistic mechanism between these two psychological mediators in users’ behaviors has not been fully elucidated. Based on the SOR model, we demonstrate that the eight GAI features (stimuli) influence infusion use (response) through the parallel mediation of perceived value and satisfaction (organism). These findings provide deeper insights into how individual cognition and attitudes jointly evolve when responding to external stimuli.

Third, these findings contribute to the literature on deep usage of GAI in online learning contexts. With the advancement of GAI technology, increasing research focuses on users’ long-term usage behaviors in online learning (Ngo et al., 2024; Holzmann et al., 2025; Liu et al., 2025). As the ultimate stage in post-adoption, infusion use represents not only the deep integration of technology into the learning process but also the latent commercial value within the education domain (Hu et al., 2024). Although infusion use has been investigated in contexts such as information technology (Sundaram et al., 2007), customer relationship management (Chen et al., 2021), and smart objects (Hu et al., 2024), its examination in online learning environments remains limited. This study addresses this gap by providing a theoretical framework for understanding GAI infusion use, thereby supplementing and enriching research on user behavior in online learning contexts.

### 6.2 Practical implications

This study offers significant practical implications for GAI researchers and developers. First, GAI service providers should adopt a long-term strategic perspective to foster users’ infusion use, thereby unlocking greater business value. As demonstrated by globally successful products like ChatGPT, Gemini, and DeepSeek, deep engagement and comprehensive feature utilization are critical to commercial success. Service providers should focus on enhancing all eight identified dimensions—intelligence, explainability, response time, integrability, accuracy, source credibility, personalization, and emotional support—while prioritizing core user needs and establishing clear strategic objectives.

Second, educational institutions and teachers should establish multi-dimensional evaluations when selecting GAI learning tools. Beyond conventional metrics like “intelligence,” criteria should also consider other factors, such as explainability and emotional support. This enables users to receive both comprehensive knowledge and psychological encouragement during challenging learning phases. Additionally, leveraging GAI to facilitate personalized learning plans can transform GAI into a valuable educational partner.

Third, developers can enhance perceived value and satisfaction by emphasizing GAI’s practical benefits and distinctive advantages. Effective strategies include implementing intelligent summaries (e.g., “I have summarized the key points of this chapter for you, saving your research time”) and generating personalized learning progress reports that help users visualize their achievement. Furthermore, incorporating empathetic interactions during complex tasks (e.g., “This question is indeed challenging. Let us tackle it step by step.”) can create a pleasant and efficient learning experience.

Finally, configuration analysis suggests that when facing technical resource constraints, developers should prioritize five key dimensions: integrability, accuracy, source credibility, personalization, and emotional support. Specific implementations may include regularly updating knowledge bases to ensure content authority and relevance, enhancing information capture and summarization capabilities, dynamic user profiles, and emotional interaction.

### 6.3 Limitations and future research

This study has several limitations. First, this study is primarily based on the sample of Chinese users, and its conclusions may be influenced by specific cultural and contextual factors. This may limit the generalizability of the findings to some extent. Future research could incorporate more diverse participants across different countries and regions. It can help validate the transferability of our findings. Furthermore, exploring how cultural differences influence the use of GAI in online learning contexts could both enhance the theoretical robustness and practical applicability of the research. Second, this study relies on cross-sectional data collected. Future studies could employ longitudinal methods to capture the dynamic nature of users’ perceptions over time. Third, while this study is grounded in the SOR model, future work could integrate other relevant theories (e.g., diffusion of innovations theory, CASA paradigm) to further enrich the understanding of factors influencing users’ infusion use.

## Data Availability

The raw data supporting the conclusions of this article will be made available by the authors, without undue reservation.

## Footnotes

^1^For detailed information about Quizlet Q-Chat, please visit https://quizlet.com/blog/meet-q-chat.

^2^For a detailed description of the report, see: https://www.questmobile.com.cn/research/report/1818126420037177346.

^3^ www.Sojump.cn
