# Supplementary material for: My digital mentor: a mixed-methods study of user-GAI interactions
Source: Front Psychol. 2025 Nov 18;16:1636480. doi: 10.3389/fpsyg.2025.1636480 (PMC12669189; doi:10.3389/fpsyg.2025.1636480)
Supplement: Supplementary file 1 [file Supplementary_file_1.docx]

My Digital Mentor: A Qualitative and Configurational Analysis in Learner-GAI Relationships

# Appendix A

## Appendix A.1

**Contextual Explanation**: Generative Artificial Intelligence (GAI) refers to dialog systems, which simulate human-like interactions with users through Natural Language Processing (NLP) and deep learning techniques. Unlike traditional rule-based or retrieval-based dialogue systems, GAI dynamically generates natural, coherent, and contextually relevant responses based on the user’s input and context, rather than relying on predefined templates or fixed database answers. Examples of GAI products include LLaMA, ChatGPT, Bard, DeepSeek, ERNIE Bot, Tencent Hunyuan, Doubao, Kimi, and others.

Interview questions:

1. How would you define online learning?

2. In your context, what are your primary uses of GAI in learning scenarios? How frequently do you use it? Please describe the process in detail.

3. Which GAI products have you used in learning scenarios? What are the similarities and differences?

4. What do you perceive as the differences between traditional offline learning, online learning, and the use of GAI?

5. What do you think will be the future trends in online learning following the emergence of GAI?

6. What do you think are the most important factors you consider when using GAI as a learning tool? How do these factors influence your usage?

7. Have you encountered any challenges or limitations when using GAI in learning scenarios?

8. In what ways would you like to see GAI improved to better meet your needs?

9. Aside from the abovementioned factors, what other factors in your life influence your use of GAI as a learning tool?

10. Would you be interested in exploring GAI's capabilities further to fully utilize them in your learning? Why or why not?

## Appendix A.2

**Table A1** Selected quotes by participants (key constructs)

| Third order categories | Second order categories | Selected Quotes | Definition |
| --- | --- | --- | --- |
| Intelligence | Task processing capability | “I find that GAI performs exceptionally well when addressing general inquiries. For example, if I ask it to write a paragraph about the future outlook and summary of intelligent transportation, it can just express that in a more concise and then more fluent paragraph. Similarly, when I ask for suggestions regarding final exam preparation, it provides comprehensive recommendations, including detailed timelines and task allocations, which I find highly effective.” (P1)  “The traditional search engine, it has no way to help me analyze what the translation is like. It can only help me locate the translation that I think is more official and correct, but it can't help me analyze the highlights of this translation. Therefore, this kind of generated copy will be more human than the traditional search engine, which is in line with the needs of humanities and social sciences like us.”(P11) | GAI is goal-oriented, capable of adapting to conversational contexts, utilizes natural language for user interaction, and demonstrates the ability to deliver efficient responses during service engagements. |
|  | Memory continuity | “Take Kimi as an example. Initially, it could only assist with basic tasks like summarizing academic papers. Now, it has evolved to handle more comprehensive functions including PPT creation, image analysis, and chart interpretation. Furthermore, its response quality has significantly improved - while previous answers typically contained 4-5 key points, it now provides more extensive and detailed analyses. This enhancement likely stems from improvements in both its information processing capabilities and web search functionalities.” (P3)  “Because I think ChatGPT records all my previous conversations, I think its answer will be more professional and more in line with my heart.” (P6) |  |
|  | Self learning | “Sometimes I feel that this answer is relatively rigid, but after my repeated questions, it may indeed give a more diverse answer.” (P8)  “I think this AI, if you can learn something according to your things, enhance its ability, and then output better results. It will give me some better feedback based on my original conversation.” (P15) |  |
|  | Understanding capability | “I think its understanding ability is limited. The more information I have to give it, the more my feedback will meet my requirements. If I just say to give it a simple one, for example, help me write a paper named XX, it will basically not meet my requirements.” (P4)  “I use Doubao in oral English practice, which is only relatively situational, but not anthropomorphic enough. A real person has a lot of emotions, and everything he may say is unpredictable, so a real person will combine his mood, mood and state at that time and want to express his meaning more, and then any answer may be different. AI I think it is very rigid. ” (P25) |  |
| Explainability | Need for process explanation | “I place significant emphasis on the reasoning process behind the answers, so I consistently request ChatGPT and other AI systems to provide detailed derivations. This approach serves two purposes: first, it allows me to verify the accuracy of the AI’s responses, as AI systems are prone to errors; second, it helps me identify potential issues such as plagiarism or other inconsistencies.” (P6)  “In most cases, AI just gives you a direct answer, and the reference content will be shorter. So I hope it will expand in process description. Sometimes when I ask a question, it may take me many times to get what I want. I hope that it will cover this process from the beginning.” (P8) | GAI's capability to present its underlying logic and decision-making processes for users to understand. |
|  | Thinking process presentation | “I would hope that it can be said that I made a comparison between the official translation and my translation, or what theory it was based on, and then made these summaries.” (P9)  “AI can give more detailed content; this thinking process is a process for me to recognize it and learn.” (P23) |  |
| Response time | Problem-solving timeliness | “Watching a video or searching for it, it's possible that someone else is experiencing a problem like this and he hasn't posted it online. Assuming that there is no one around to help you solve the problem, or if you can't find it at this time, the probability is that the problem will be shelved. But if you have AI, it can't completely help you solve the problem, but it can give you some direction, or give you some ideas, and then it may be able to help you solve it better.” (P2)  “As opposed to AI, if you find a problem with a library on GitHub, then first you have to go and raise an issue and give it to someone else, and then they don't necessarily get back to you. Or you go to the issue and search for it, but odds are it's not going to have the same problem that you're having.” (P21) | Timeliness of GAI responses to users. |
|  | Save time | “One of the big reasons that prompted me to use ChatGPT is that it will be more convenient. It will take me more time to discuss with my classmates or ask the teacher.” (P6)  "If you use MOOC or bilibili, or some other large and well-known platforms, in fact, one thing they have in common is that you need to watch videos, which may require some investment in your time cost. But instead, using the generative AI software, it can give you a result in a few seconds. More of this kind of text impact is different from the visual video impact, because the video may bring you a sense of picture, or you can immerse yourself in it to learn something in more detail. But it doesn't necessarily mean that I have enough time to study. Therefore, the current one is that teachers don't have much private space, and generative software provides us with a cost-saving and energy-saving, so we don't have to search on various video platforms. " (P14) |  |
| Integrability | Summarize | “The AI will also summarize and categorize it for you. If you go to the online class or go elsewhere to search for some short videos, it may just give you an explanation of the process, but in the process of the explanation it does not give you to generate some text things. Like AI, it directly gives you all the layout, and then, the summary is also all summarized for you.” (P5)  “Learning these things (financial management), the basic concepts, and so on, in fact, sometimes Baidu can also find. But AI is equivalent to sifting through the useless information for me, what ads, what nonsense, and directly give me a clearer answer. At the same time, it can also have an interactive dialog, and I'm equivalent to being able to make that thing more coherent (learn) from start to finish.” (P24) | GAI effectively facilitates the ability to combine information from different sources to respond to user problems. |
|  | Information integration | “I think if I use a search engine, it will find too much content, and many of them are not what I want. If you use AI, it is to give a more appropriate answer directly according to your question. I think it is more convenient, so you don't have to screen it yourself.” (P1)  “If you go to online classes, or go to other places to search for some short videos, maybe it just gives you an explanation process. But in the process of explanation, it can't generate some words for you. If it is AI, it will be typeset directly for you, and then it will be summarized for you.” (P21) |  |
| Accuracy | Find a wrong answer | “I feel that every time I check it, I am afraid that what I have found is wrong, and if I use it, it will have any impact. I hope the answers and data of GAI can be more accurate and reduce errors.” (P1) | GAI's capability to provide up-to-date and relevant information that responds to the user's intended goals. |
|  | Incorrect information | “I feel that I am afraid that what I have found is wrong every time. If I use it, will it have any impact? It is definitely to hope that its answers and data can be more accurate, that is, to reduce mistakes.” (P1)  “A lot of AI answers, it retrieved a lot of information and then generated its understanding. There will be a problem, that is, after it is searched, and in the process of generating, did it add something incorrect ...... This thing is not what it searched, but what it randomly added in. Or it is based on certain rules, or based on its algorithmic logic, and then it added a fake thing. So this differentiation between real and fake is also a problem, especially when it comes to something where you need very real data and you can't have any fake data, and this time he will give you some fake data, and that will be very troublesome.” (P7) |  |
|  | Information timeliness | “There are some official data or real-time information, and I don't believe 100% of the answers it gives me.” (P9)  “The data set used in its model is January 2024. Because I asked. For example, there are some frameworks in writing code, which are actually released in the summer (2024) and at that time in July and August. Then you ask the code of the AI framework, which is actually not available. Then it is definitely unknown to the cutting-edge articles.” (P21) |  |
| Source credibility | Expertise | “There is something that involves more official data or more real-time information, and I don't trust 100% of the answers that it gives me. When I used it today (trying to find) a conceptual noun, I asked it about it and then it said that the coordinator is not currently in a fixed position in the International area. But actually, that coordinator now is China.” (P9)  “Carbon offsetting, it's a more specialized term, I want to search which company this affects more, then I want to be aware of some news or something like that. Sometimes there's no corresponding news for that. Then ChatGPT makes up its own and then gives you something that's a very generalized one. Then I don't trust it, and I ask it, what's your news source? It gives you a link to a news publisher like CNN or something like that, and then when you click on it, it comes right up with a page that's 404, there's no such news.” (P17) | Trustworthiness and expertise of GAI information sources. |
|  | Trustworthiness | “My evaluation of its authenticity depends on cross-verification. When discrepancies emerge between its generative results and my Baidu search findings, I often reach an epistemic impasse where neither source can be conclusively validated.” (P14)  “I think about the authenticity of GAI, for example, if the results it pushed me are different from those I searched Baidu, I may not be able to judge which information is true” (P21). |  |
| Personalization | Specific needs | “If you have some very specific needs, you may still have to do it on your own. The AI may be less able to understand your particularly specific needs.” (P13)  “Students ask some broad questions, so he will ask AI, because AI can give him some formulaic answers. However, if you ask some small, personal and special questions, maybe he will not ask AI, but choose to ask teachers or classmates. Because in this case, AI can't give him a specific answer.” (P19) | GAI provides customized services to users based on their needs, preferences, and intent. |
|  | User preference | “I may send all the text I need to it (GAI), and then select my favorite teachers and my favorite teaching methods online and transmit them to it. I don't know if it can combine these, combine my favorite knowledge with my favorite expression, my favorite language description, etc., and give me all the feedback with the feeling of an objective reality.” (P8)  “Because in fact, I think everyone can use AI now. But it has certain uniqueness. For example, what I expect more is that it can target different people, or it can collect different interests and hobbies of different users. It directly pushes relevant content to you, which I think is also better.” (P21) |  |
|  | Professional advice | “Because many of my questions will be very professional, I would prefer to have such a personalized and professional AI.” (P6)  “AI can provide some solid and professional suggestions according to different disciplines or different majors. I think this is the most important. If what it gives you is all that kind of vague things, this is definitely not acceptable. Because the purpose of using it is to tend to get professional guidance or advice from it.” (P14) |  |
| Emotional support | Empathy | “ChatGPT, I think the feedback it gave me is quite comprehensive. That is, it talked about many aspects. For example, I told it that I now feel depressed because a task was not finished, or what other circumstances I encountered, and then I feel that it is very difficult to solve. It will calm you down first, and then give you some new suggestions. For example, what does it think you should do? Or give you some emotional support.” （P2）  “The AI has a pretty positive view of things. If you're sometimes busy with finals and want to go crazy, say something to it and it'll even help channel you.” (P3) | GAI communicates empathy, emotional validation, and encouragement to users. |
|  | Emotional venting | “AI doesn't have to comfort me, it just listens to me.” (P10)  “Most people don't necessarily need to go to a consultant to help him solve something. Sometimes what most people need is an outlet. Then this kind of consultation between us and others, after all, the resources of psychological counselors are limited. I think AI can make up for this part, and I think it is also very good.” (P26) |  |
|  | Supportive | “I think AI is completely different from people's feedback. Maybe people will tell you that the news industry is not very good at finding a job, and the employment situation is not very optimistic now. But the answer given by AI is relatively neutral, and there is still a way out for the news industry. That is, it may just give me some vitality, unlike most people who say the problem to death.” (P16)  “I would feel that the AI is very positive, and the replies it can often give to me are very supportive and constructive, so I would be happier talking to it. It doesn't deny me anything. The most it will say is that it's not OK for you to be like this and it will say differently that this is risky.” (P16) |  |
| Perceived value | Time spent pays off | “I feel that AI reduces some labor costs. In the past, the search software was often used by students in junior and senior high schools. In fact, that class is about explaining the answers. If it usually comes with a video, that kind of class just needs to recruit some part-time college students to record it, and then help them explain it before it can be posted on the website. But now it is possible to use this technology to generate some answers according to this topic, and then explain the answers in text. AI can solve this problem without the cost of manpower, which I think is very cost-effective.” (P2)  “I have to sift through the answers myself and choose the one that I think is more suitable for me. Because although I gave it a framework, it is actually quite big. I may still need to spend time identifying and analyzing right and wrong. However, compared with Baidu, time is less, and the answer can give me some inspiration.” (P10) | Users’ overall assessment of the utility of GAI based on perceptions of what is received and what is given |
| Satisfaction | The content is good | “I think if you ask a common question, it answers very well.” (P1)  “AI, like Doubao, is definitely helpful, because it will make me more and more fluent, and then the expression will become more and more natural, and the response will get better and better with all aspects.” (P25) | The extent to which users perceive that the GAI meets their needs after interacting with it. |

# Appendix B

**Table B1** Measurement items

| Constructs | Items | References |
| --- | --- | --- |
| Intelligence | INTEL1: GAI can communicate with me in an understandable manner.  INTEL2: GAI can find and process the necessary information for completing the tasks.  INTEL3: GAI can provide me with a useful answer.  INTEL4: GAI can understand my instructions.  INTEL5: GAI shows professional knowledge and capability in the process of interaction. | Mehmood et al. (2024) and D. Liu et al. (2023) |
| Explainability | EXP1: I am clear of GAI’s reasoning process.  EXP2: It is easy for me to see how GAI generates its responses.  EXP3: I can’t understand how GAI performs its job. [R]  EXP4: It is easy for me to understand the inner workings of GAI.  EXP5: I can understand why and how GAI makes the recommendations. | Darban (2024) |
| Response time | RT1: GAI’s response time is fast.  RT2: The response time of the GAI is acceptable.  RT3: It does not take too long for the GAI to respond to my requests. | Y.-l. Liu et al. (2023) |
| Integrability | INTEG1: I think the GAI has the ability to integrate data from different fields effectively.  INTEG2: I think the GAI has the ability to integrate information from different places effectively.  INTEG3: I think the GAI has the ability to combine data from different areas effectively. | Chen et al. (2025) |
| Accuracy | ACC1: GAI produces accurate information.  ACC2: GAI produces complete information.  ACC3: Information provided by GAI is free of errors.  ACC4: GAI intentionally gives me false information. [R] | Yuan et al. (2022) and Zhou and Wu (2024) |
| Source credibility | SC1: GAI has a great amount of experience to solve my questions.  SC2: GAI has great expertise in the field of learning.  SC3: I trust that GAI provides information related to learning.  SC4: I believe GAI made a truthful claim.  SC5: The information provided by GAI related to learning is dishonest. [R] | Wilson and Baack (2023) |
| Personalization | PER1: I perceive that the content and information recommendations from GAI match my preference very well.  PER2: GAI replies with the same as I wanted.  PER3: I perceive that the content and information recommendations from GAI fit my tastes very well.  PER4: I feel like I just had a personal conversation with a sociable, knowledgeable and warm representative when I use GAI.  PER5: GAI is sensitive to my needs. | Chen, Gong, et al. (2023) and Zhu et al. (2023) |
| Emotional support | ES1: When I share my learning difficulties with GAI, it is on my side.  ES2: When I share my learning difficulties with GAI, it comforts and encourages me  ES3: When I share my learning difficulties with GAI, it listens to me when I talk about my private feelings  ES4: When I share my learning difficulties with GAI, it expresses interest and concern about my well-being | Zhang et al. (2018) |
| Perveived value | PV1: Compared to the time a traditional online learning is provided, the use of GAI in a is worthwhile to me.  PV2: Compared to the cost I need to pay, the use of GAI offers value for money.  PV3: GAI is an excellent value for my time and effort  PV4: I think it is valuable for me to spend time and effort interacting with GAI. | De Kervenoael et al. (2020) and Chen, Lu, et al. (2023) |
| Satisfaction | SAT1:I am satisfied with GAI’s service.  SAT2: GAI is a good representative for online learning.  SAT3: GAI meets my expectations. | Xu et al. (2023) |
| Infusion use | IU1: I am using all capabilities of GAI in the best fashion to help me accomplish my daily learning.  IU2: I have used GAI in the best way to support me in my daily learning.  IU3: I have optimally used GAI to support me in my daily learning.  IU4: My use of GAI in my daily learning has been integrated and incorporated at the highest level | Hu et al. (2024) |
| Note: [R] represents a reverse item | | |

**Table B2** Common method bias test according to Liang et al. (2007)

| Construct | Indicator | Substantive factor loading (R1) | ${\boldsymbol{R}\boldsymbol{1}}^{\boldsymbol{2}}$ | Method factor loading (R2) | ${\boldsymbol{R}\boldsymbol{2}}^{\boldsymbol{2}}$ |
| --- | --- | --- | --- | --- | --- |
| Intelligence | INTEL1 | 0.843 | 0.711 | -0.016 | 0.000 |
|  | INTEL2 | 0.859 | 0.738 | -0.018 | 0.000 |
|  | INTEL3 | 0.830 | 0.689 | 0.026 | 0.001 |
|  | INTEL4 | 0.850 | 0.723 | -0.006 | 0.000 |
|  | INTEL5 | 0.826 | 0.682 | 0.015 | 0.000 |
| Explainability | EXP1 | 0.857 | 0.734 | 0.045 | 0.002 |
|  | EXP2 | 0.873 | 0.762 | 0.001 | 0.000 |
|  | EXP3 | 0.884 | 0.781 | -0.033 | 0.001 |
|  | EXP4 | 0.863 | 0.745 | 0.010 | 0.000 |
|  | EXP5 | 0.871 | 0.759 | -0.022 | 0.000 |
| Response time | RT1 | 0.883 | 0.780 | 0.002 | 0.000 |
|  | RT2 | 0.873 | 0.762 | 0.002 | 0.000 |
|  | RT3 | 0.855 | 0.731 | -0.004 | 0.000 |
| Integrability | INTEG1 | 0.864 | 0.746 | -0.103 | 0.011 |
|  | INTEG2 | 0.887 | 0.787 | 0.049 | 0.002 |
|  | INTEG3 | 0.848 | 0.719 | 0.051 | 0.003 |
| Accuracy | ACC1 | 0.839 | 0.704 | 0.021 | 0.000 |
|  | ACC2 | 0.869 | 0.755 | -0.036 | 0.001 |
|  | ACC3 | 0.879 | 0.773 | -0.026 | 0.001 |
|  | ACC4 | 0.861 | 0.741 | 0.050 | 0.003 |
| Source credibility | SC1 | 0.844 | 0.712 | -0.006 | 0.000 |
|  | SC2 | 0.878 | 0.771 | -0.037 | 0.001 |
|  | SC3 | 0.877 | 0.769 | 0.009 | 0.000 |
|  | SC4 | 0.847 | 0.717 | 0.045 | 0.002 |
|  | SC5 | 0.860 | 0.740 | -0.020 | 0.000 |
| Personalization | PER1 | 0.837 | 0.701 | 0.042 | 0.002 |
|  | PER2 | 0.846 | 0.716 | -0.039 | 0.002 |
|  | PER3 | 0.861 | 0.741 | 0.040 | 0.002 |
|  | PER4 | 0.878 | 0.771 | -0.022 | 0.000 |
|  | PER5 | 0.841 | 0.707 | -0.021 | 0.000 |
| Emotional support | ES1 | 0.838 | 0.702 | 0.002 | 0.000 |
|  | ES2 | 0.870 | 0.757 | 0.004 | 0.000 |
|  | ES3 | 0.862 | 0.743 | -0.025 | 0.001 |
|  | ES4 | 0.846 | 0.716 | 0.019 | 0.000 |
| Perceived value | PV1 | 0.866 | 0.750 | 0.024 | 0.001 |
|  | PV2 | 0.890 | 0.792 | 0.005 | 0.000 |
|  | PV3 | 0.885 | 0.783 | -0.085 | 0.007 |
|  | PV4 | 0.874 | 0.764 | 0.056 | 0.003 |
| Satisfaction | SAT1 | 0.906 | 0.821 | 0.106 | 0.011 |
|  | SAT2 | 0.913 | 0.834 | -0.083 | 0.007 |
|  | SAT2 | 0.908 | 0.824 | -0.024 | 0.001 |
| Infusion use | IU1 | 0.850 | 0.723 | 0.025 | 0.001 |
|  | IU2 | 0.860 | 0.740 | -0.022 | 0.000 |
|  | IU3 | 0.856 | 0.733 | 0.018 | 0.000 |
|  | IU4 | 0.857 | 0.734 | -0.020 | 0.000 |
| Average |  |  | 0.746 |  | 0.002 |

# References

Chen, H., Wang, P., & Hao, S. (2025). AI in the spotlight: The impact of artificial intelligence disclosure on user engagement in short-form videos. *Computers in Human Behavior*, *162*, 108448. <https://doi.org//10.1016/j.chb.2024.108448>

Chen, Q., Gong, Y., & Lu, Y. (2023). User experience of digital voice assistant: Conceptualization and measurement. *Acm Transactions on Computer-Human Interaction*, *31*(01), 1-35. <https://doi.org/10.1145/3622782>

Chen, Q., Lu, Y., Gong, Y., & Xiong, J. (2023). Can AI chatbots help retain customers? Impact of AI service quality on customer loyalty. *Internet Research*, *33*(06), 2205-2243. <https://doi.org/10.1108/INTR-09-2021-0686>

Darban, M. (2024). Navigating virtual teams in generative AI-led learning: The moderation of team perceived virtuality. *Education and Information Technologies*, 1-24. <https://doi.org/10.1007/s10639-024-12681-4>

De Kervenoael, R., Hasan, R., Schwob, A., & Goh, E. (2020). Leveraging human-robot interaction in hospitality services: Incorporating the role of perceived value, empathy, and information sharing into visitors' intentions to use social robots. *Tourism Management*, *78*, 104042, Article 104042. <https://doi.org/10.1016/j.tourman.2019.104042>

Hu, Q., Pan, Z., Lu, Y., & Gupta, S. (2024). How does material adaptivity of smart objects shape infusion use? The pivot role of social embeddedness. *Internet Research*, *34*(04), 1219-1248. <https://doi.org/10.1108/INTR-04-2022-0253>

Liang, H. G., Saraf, N., Hu, Q., & Xue, Y. J. (2007). Assimilation of enterprise systems: The effect of institutional pressures and the mediating role of top management. *Mis Quarterly*, *31*(01), 59-87. <https://doi.org/10.2307/25148781>

Liu, D., Lv, Y., & Huang, W. (2023). How do consumers react to chatbots' humorous emojis in service failures. *Technology in Society*, *73*, 102244.

Liu, Y.-l., Hu, B., Yan, W., & Lin, Z. (2023). Can chatbots satisfy me? A mixed-method comparative study of satisfaction with task-oriented chatbots in mainland China and Hong Kong. *Computers in Human Behavior*, *143*, 107716. <https://doi.org/10.1016/j.chb.2023.107716>

Mehmood, K., Kautish, P., & Shah, T. R. (2024). Embracing digital companions: Unveiling customer engagement with anthropomorphic AI service robots in cross-cultural context. *Journal of Retailing and Consumer Services*, *79*, 103825. <https://doi.org/10.1016/j.jretconser.2024.103825>

Wilson, R. T., & Baack, D. W. (2023). How the credibility of places affects the processing of advertising claims: A partial test of the B2B communication effects model. *Journal of Business Research*, *168*, 114238. <https://doi.org/10.1016/j.jbusres.2023.114238>

Xu, Y., Niu, N., & Zhao, Z. (2023). Dissecting the mixed effects of human-customer service chatbot interaction on customer satisfaction: An explanation from temporal and conversational cues. *Journal of Retailing and Consumer Services*, *74*, 103417.

Yuan, C., Zhang, C., & Wang, S. (2022). Social anxiety as a moderator in consumer willingness to accept AI assistants based on utilitarian and hedonic values. *Journal of Retailing and Consumer Services*, *65*, 102878. <https://doi.org/10.1016/j.jretconser.2021.102878>

Zhang, X., Liu, S., Chen, X., Wang, L., Gao, B. J., & Zhu, Q. (2018). Health information privacy concerns, antecedents, and information disclosure intention in online health communities. *Information & Management*, *55*(04), 482-493. <https://doi.org/10.1016/j.im.2017.11.003>

Zhou, T., & Wu, X. (2024). Examining user migration intention from social Q&A communities to generative AI. *Humanities and Social Sciences Communications*, *11*(01), 1-10. <https://doi.org/10.1057/s41599-024-03540-1>

Zhu, Y., Zhang, R., Zou, Y., & Jin, D. (2023). Investigating customers’ responses to artificial intelligence chatbots in online travel agencies: The moderating role of product familiarity. *Journal of Hospitality and Tourism Technology*, *14*(2), 208-224. <https://doi.org/10.1108/JHTT-02-2022-0041>
